# Circular RNAs: clinical relevance in cancer

**DOI:** 10.18632/oncotarget.22846

**Published:** 2017-12-01

**Authors:** Zhijie Xu, Yuanliang Yan, Shuangshuang Zeng, Shuang Dai, Xi Chen, Jie Wei, Zhicheng Gong

**Affiliations:** ^1^ Department of Pathology, Xiangya Hospital, Central South University, Changsha 410008, China; ^2^ Department of Pathology, School of Basic Medicine, Central South University, Changsha 410008, China; ^3^ Department of Pharmacy, Xiangya Hospital, Central South University, Changsha 410008, China; ^4^ Institute of Hospital Pharmacy, Central South University, Changsha 410008, China

**Keywords:** circular RNAs, miRNA sponges, gene expression regulation, biomarkers, cancer

## Abstract

Circular RNAs, as recently discovered new endogenous non-coding RNAs, are important gene modulators with critical roles in tumor initiation and malignant progression. With the development of RNA sequencing and microarray technologies, numerous of functional circRNAs have been identified in cancerous tissues and cell lines. Mechanistically, circRNAs function as miRNA sponges, miRNA reservoirs or parental gene expression regulators. In this review, we discuss the properties and functions of circRNAs and their clinical implication as promising biomarkers for cancer research. Moreover, some emerging fields, such as exosome-loaded and immune response-associated circRNAs, are also discussed, suggesting novel insights into the carcinogenesis and therapy associated with these molecules.

## INTRODUCTION

Following long non-coding RNAs (lncRNA) and microRNAs (miRNAs), circular RNAs (circRNAs) are a novel class of non-coding RNAs (ncRNAs), representing a new paradigm of gene regulation in many biological processes, such as proliferation, senescence and apoptosis [1, 2]. As early as the 1980s, circRNAs were initially discovered in RNA viruses, such as plant viroids. Unfortunately, reflecting their low abundance, circRNAs were originally considered as splicing byproducts or background noise [3, 4]. Currently, thousands of circRNAs have been identified and characterized with the development of high throughput RNA sequencing (RNA-seq) technologies and powerful computational analyses, and the expression level of some circRNAs is significantly higher than that of their corresponding linear isoforms from the same genes [5, 6].

To date, emerging studies have confirmed that circRNAs are endogenous, abundant and conserved in mammalian cells [7, 8]. The modulation of circRNAs on gene expression plays a significant role in various physiological and pathological procedures, particularly carcinogenesis [9, 10]. Moreover, subsequent reports have established the circRNAs-miRNAs-mRNA signaling axis, which participates in the occurrence and development of various cancers, such as oral carcinomas [11], hypopharyngeal cell carcinoma [12], osteosarcoma [13], etc. Thus, circRNAs show great potential as promising clinical biomarkers and therapeutic targets for cancers.

In this review, we primarily focus on the functions and potential roles of circRNAs in cancer research and therapy. In addition, we discuss some inspiring areas for future research, which may influence potential clinical implications, specifically serving as therapeutic targets. Finally, some algorithms detecting circRNAs from RNA-seq data are also preliminarily summarized.

## CHARACTERISTICS OF circRNAs

**Figure 1:**
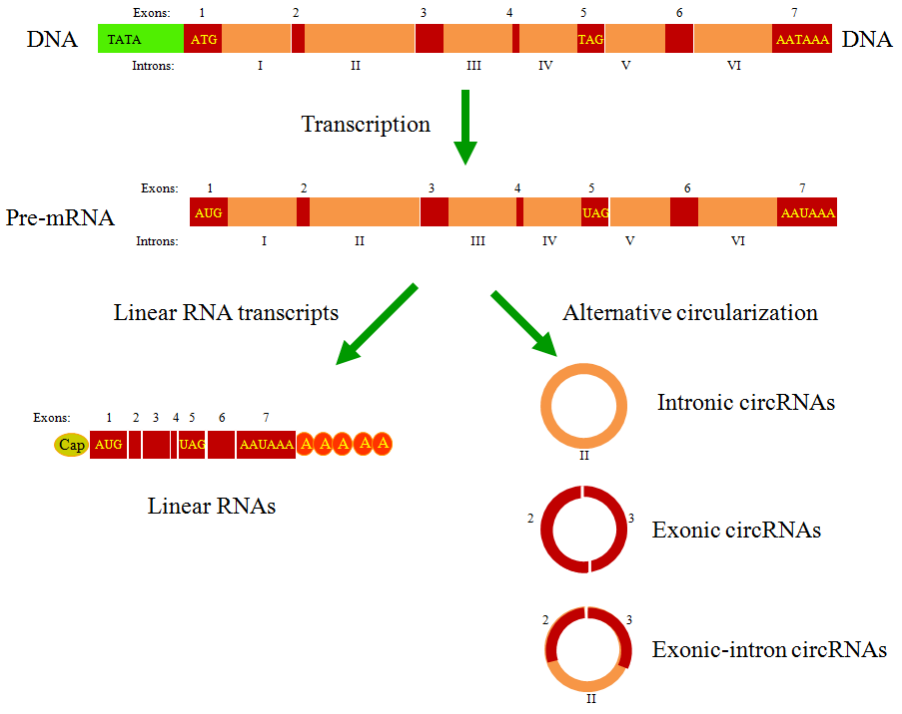
The types of circRNAs generated from the pre-mRNA by backsplicing Three main types of circRNAs were recently identified: intronic circRNAs, exonic circRNAs and exonic-intron circRNAs. Studies have demonstrated that circRNAs are most likely generated co-transcriptionally from the same gene locus in competition with the formation of canonical linear RNA transcripts, a phenomenon called alternative circularization.

**Figure 2:**
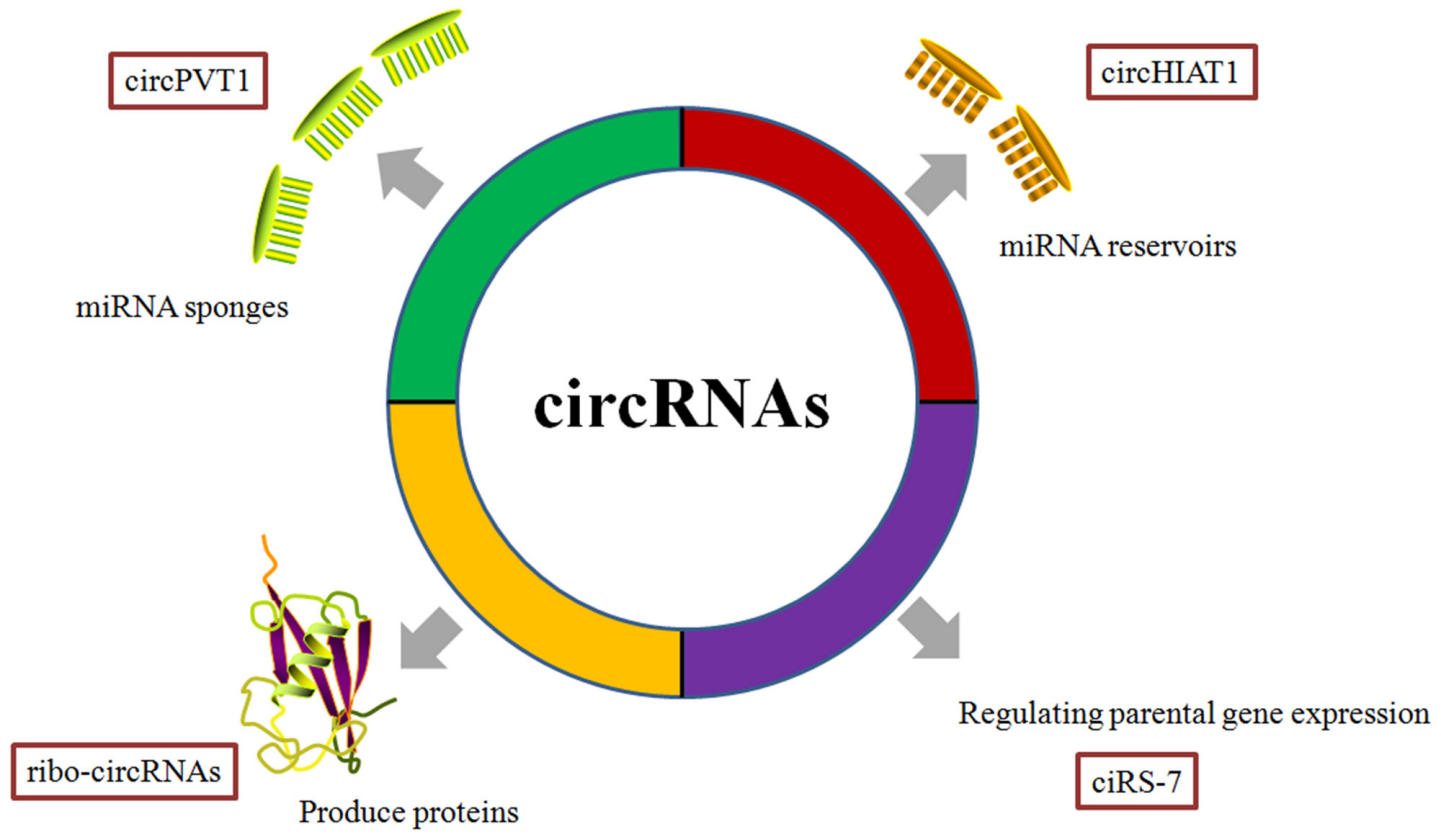
Functions of circRNAs determined through cancer research CircRNAs serve as miRNA sponges/reservoirs or parental gene expression regulators involved in the onset and progression of cancers. Although the complete function of circRNAs in cancer biology remains unidentified clearly, several probable mechanisms have been reported so far. In general, a well-known function of circRNAs is of miRNA regulation through sponging miRNAs, if circRNA contains the corresponding miRNA binding sites. For example, circPVT1 promotes gastric cancers progression via acting as miR-125 sponge. Surprisingly, a specific circRNAs with corresponding miRNA binding sites, like cytoplasmic circHIAT1, serves as a miRNA reservoir, further stabilizing the miR-195-5p/29a-3p/29c-3p in renal cell carcinoma. In addition, some circRNAs, like ciRS-7/CDR1-AS, regulate parental genes through direct interactions. CiRS-7 is reported to enhance the CDR1 expression by interacting and stabilizing the sense CDR1 mRNA.

Unlike linear RNAs, circRNAs form a covalently closed continuous loop without free caps and poly(A) tails [14-16] (Figure 1). Reflecting their unique structure, circRNAs have several noteworthy properties. First, the lack of exposed 3’ and 5’ terminals makes circRNAs more stable and less susceptible to degradation by ribonuclease R (RNase R) or other exonucleases. Studies have indicated that RNase R could degrade any linear RNA, resulting in the enrichment of pure circRNAs in RNA samples. After treatment with RNase R, numerous differentially expressed circRNAs could be precisely identified in tumor tissues relative to adjacent nonneoplastic tissues [17, 18]. Second, based on differences in backsplicing mechanisms, circRNAs could be divided into three types: intronic circRNAs, exonic circRNAs and exonic-intron circRNAs [19]. Quaking (QKI), an RNA-binding protein regulated in cancer cells during the epithelial-mesenchymal transition (EMT), promotes RNA backsplicing, producing mature circRNA [20]. Third, the majority of circRNAs are evolutionarily conserved. Thomas et al. confirmed an decreased SNP (single nucleotide polymorphism) density in circRNA sequences, particularly the miRNA-binding sites [21]. Fourth, most circRNAs are located in the cytoplasm, whereas the circRNAs from introns are abundant in the nucleus [22]. Nuclear circRNAs regulate RNA transcription by binding the cognate DNA locus [23] or RNA polymerase II [24]. Cytoplasmic circRNAs primarily function as miRNA sponges that inhibit miRNA expression and weaken the translation suppression on the corresponding target molecules [25] (Figure 2). Through paired-end RNA-seq on samples obtained from three ovarian cancer patients, Ahmed et al. showed that thousands of circRNA isoforms have predicted miRNA response elements (MREs). Over-expression of these circRNAs could effectively regulate the downstream target genes by sponging the miRNAs involved in carcinogenesis [26]. However, another study showed that cytoplasmic circHIAT1 could also serve as a miRNA reservoir, further stabilizing the miRNA expression in renal cell carcinoma (RCC) [27] (Figure 2). These contradictory findings indicate that further studies are needed to explore circRNA-miRNA networks and obtain a comprehensive understanding of the properties of circRNAs in cancer biology. Fifth, the expression profiles showed that these molecules are expressed in a tissue- or cancer-specific manner [28]. The database CircNet was used to successfully illustrate this profile across human transcriptome samples [29]. Last, it is clear that as potential tumor markers, circRNAs are sensitive and specific. Chen et al. reported that the sensitivity and specificity of has_circ_0000190, a downregulated circRNA in gastric cancer, is much better than that of carcino-embryonic antigen (CEA) and CA19-9, two commonly used biomarkers in cancer diagnoses [30]. Taken together, the abovementioned characteristics of circRNAs suggest that these molecules are promising molecular biomarkers and novel therapeutic targets for future drugs.

Additionally, although circRNAs were originally defined as a class of ncRNAs, newly published studies revealed that several circRNAs could function as mRNAs that produce functional proteins, indicating cap-dependent translation [31-33] (Figure 2). By screening the open reading frames (ORFs) using ribosome footprinting (RFP) datasets, Pamudurti et al. showed ribosomes associated circRNAs (ribo-circRNAs) have coding potential in *Drosophila* S2 cells. Further mass spectrometry analysis demonstrated that the encoded proteins from ribo-circRNA isoforms could be modulated by the transcription factor forkhead box protein O (FOXO) and starvation [34]. Similarly, through polysome profiling analysis, computational prediction and mass spectrometry in human embryonic kidney 293 cells and cervical cancer HeLa cells, Yang et al. identified hundreds of endogenous circRNAs with translation abilities, which were dependent on the motifs of N6-methyladenosine (m^6^A) in the sequence of the molecules, and the initiation factor eIF4G2 (eukaryotic translation initiation factor 4 gamma 2) and m^6^A reader YTHDF3 (YTH m^6^A RNA-binding protein 3) were shown to drive this m^6^A-mediated translation [35]. Legnini’s group provided another example of protein-coding circRNA, Circ-ZNF609. These studies showed that Circ-ZNF609 contains an ORF, similar to its parent linear transcript, and could be translated into protein in a cap-independent manner [36]. Further studies are needed to determine whether circRNA translation responds to different stress stimuli or cancer-related signals, eventually leading to tumorigenesis.

## circRNAs ACT AS PROMISING TARGETS FOR CANCER BIOLOGY

In tumor biology, it is important to identify new potential biomarkers, which are differentially expressed between cancers and normal tissues. A number of studies have demonstrated that circRNAs are closely associated with cancer initiation and development [37-39] (Table 1). In addition, circRNAs could be secreted into human body fluids, such as saliva [40] and blood [41], or enriched in exosomes [42], emerging as effective biomarkers for the detection of cancers. The aberrant circRNAs discovered in cancers are discussed in detail below.

**Table 1:** CircRNA expression profiles in cancer tissues and cell lines

Cancers	CircRNAs	Expression level	Samples	Mechanisms	Refs
Oral carcinoma	circRNA_100290	Up	Tissues	MiR-29 sponge	[11]
Osteosarcoma	circUBAP2	Up	Tissues	MiR-143 sponge	[13]
Thyroid carcinoma	hsa_circRNA_100395	Down	Tissues	Interaction with miR-141-3p/200a-3p	[17]
Renal carcinoma	circHIAT1	Down	Tissues	MiR-195-5p/29a-3p/29c-3p sponge	[27]
Glioma	cZNF292	Up	Cells	Promoting Wnt/β-catenin signaling	[47]
	ciRS-7	Down	Tissues/Cells	MiR-671-5p targets	[49]
Lung cancer	circRNA_100876	Up	Tissues	–	[50]
	hsa_circ_0013958	Up	tissues/Cells/Plasma	MiR-134 sponge	[51]
	cir-ITCH	Down	Tissues	Inhibiting Wnt/β-catenin signaling	[52]
Hepatic carcinoma	circZKSCAN1	Down	Tissues	Regulating cancer-related pathways	[53]
	hsa_circ_0005075	Up	Tissues	MiR-23b-5p sponge	[56]
	hsa_circ_0001649	Down	Tissues	–	[57]
	circRNA_100338	Up	Tissues/Cells	MiR-141-3p sponge	[58]
	hsa_circ_0004018	Down	Tissues	Interaction with miR-30e-5p/626	[59]
	ciRS-7	Up		Targeting miR-7	[60]
	ciRS-7	No significance	Tissues	–	[61]
Colorectal cancer	circ-BANP	Up	Tissues	Promoting Akt phosphorylation	[65]
	hsa_circ_0000069	Up	Tissues	–	[66]
	circCCDC66	Up	Tissues	Regulating a subset of oncogenes	[67]
	circRNA_001569	Up	Tissues	MiR-145 sponge	[68]
	has_circ_0020397	Up	Cells	Promoting the miR-138 target genes	[69]
	hsa_circ_001988	Down	Tissues	–	[70]
Gastric cancer	hsa_circ_0000190	Down	Tissues	–	[30]
	circRNA-0026	Down	Tissues	Regulating cancer-associated miRNAs	[76]
	circPVT1	Up	Tissues	MiR-125 family sponge	[77]
	hsa_circ_0000096	Down	Tissues	Inhibiting cyclin D1, CDK6 and MMP2/9	[78]
	circRNA_100269	Down	Tissues/Cells	Targeting miR-630	[79]
	hsa_circ_0001895	Down	Tissues/Cells	–	[80]
	has_circ_0003159	Down	Tissues	–	[81]
Leukemia	f-circRNAs	Up	Cells	–	[86]
	hsa_circ_0004277	Down	Tissues	–	[88]
Bladder carcinoma	circRNA MYLK	Up	Tissues	Binding competitively miRNA-29a family	[90]
	circTCF25	Up	Tissues	miR-103a-3p/107 sponge	[91]
Esophageal carcinoma	has_circ_0067934	Up	Tissues/Cells	–	[93]
Breast cancer	has_circ_100219	Down	Tissues	Sequestering miRNAs	[96]
	circDENND4C	Up	Cells	HIF1α dependent	[97]
	circ-ABCB10	Up	Tissues	MiR-1271 sponge	[98]
	circ-Amotl1	Up	Tissues/Cells	inducing c-Myc nuclear translocation	[99]

## circRNAs AND GLIOMAS

**Figure 3:**
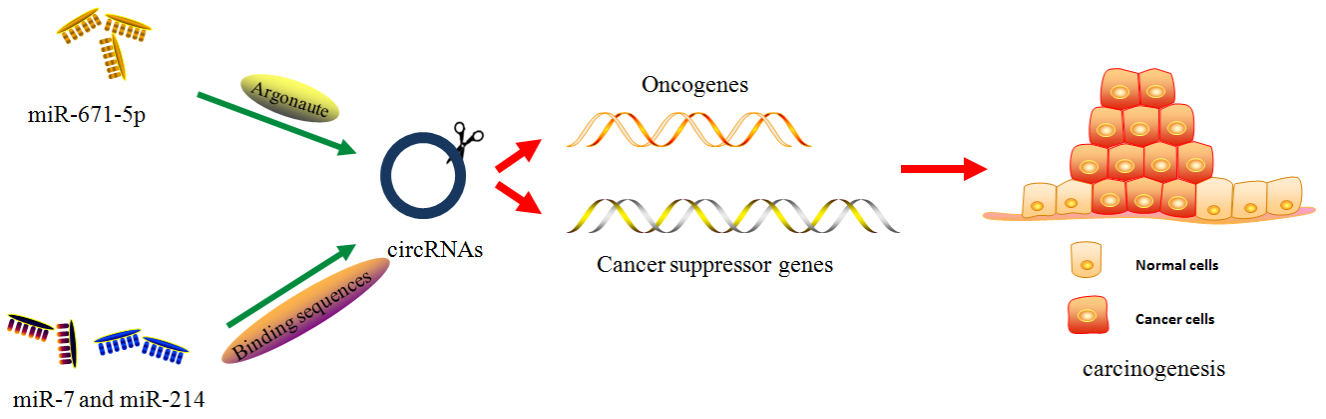
The miRNA-mediated cirRNA degradation Emerging studies have demonstrated miRNA could mediate the cirRNA degradation via Argonaute-mediated signaling or circRNA-binding sequences, further to regulating the downstream cancer-associated genes, oncogenes or cancer suppressor genes. The detailed mechanisms are well explained in the relevant sections.

Glioblastoma or glioblastoma multiform (GBM) is a highly aggressive and invasive astrocytic cell neoplasm, with malignancy grades and histological subtypes. After initial diagnosis, the median survival time of glioma patients is approximately 12-15 months [43]. To inhibit disease progression and improve the therapeutic effect of patients with GBM, it is imperative to screen for more efficient biomarkers involved in glioma biological behaviors. Studies on ncRNAs, including lncRNAs [44] and circRNAs [45], have recently become a hotspot topic that could provide insight into gliomagenesis. Using the UROBORUS tool to analyze the RNA-seq data from 27 gliomas and 19 normal brain samples, studies have revealed more than 476 circRNAs differentially expressed in gliomas and control samples [46]. Further studies have demonstrated that cZNF292, an oncogenic circRNA, is significantly upregulated in human glioma cell lines U87 and U251. Targeted silencing of cZNF292 using short interfering RNA (siRNA) could induce cell cycle arrest via the Wnt/β-catenin signaling pathway, resulting in proliferation inhibition in glioma cells [47]. In addition, some circRNAs, such as ciRS-7/CDR1-AS (cerebellar degeneration-related protein 1 antisense), could stabilize and promote the expression of parental genes through direct interactions [48] (Figure 2). In gliomas, the upregulation of oncomiR miR-671-5p induced Argonaute-mediated ciRS-7 degradation. The overexpression of ciRS-7 positively regulated the expression of CDR1, abrogating the oncogenic function of miR-671-5p in glioma biopsies and cell lines [49] (Figure 3). Based on these findings, circRNAs are proposed to play key roles in the modification of glioma pathological profiles.

## circRNAs AND LUNG CANCERS

The clinical application of circRNAs as diagnostic and prognostic indicators for lung cancers is increasingly investigated. Through the analysis of the circRNA expression features in lung cancer and adjacent normal tissues using qPCR, Yao et al. showed that circRNA_100876, encoded by RNF121 (ring finger protein 121) gene, is significantly upregulated in lung cancer samples, and Kaplan-Meier survival analysis revealed that patients with high expression levels of circRNA_100876 showed shorter overall survival times [50]. Similarly, using microarrays to screen the tumour-specific circRNA candidates in lung adenocarcinoma patients, Zhu et al. identified a significantly upregulated circRNA, hsa_circ_0013958, and found that hsa_circ_0013958 overexpression could promote tumor progression and apoptosis inhibition in lung adenocarcinoma via sponging miR-134 [51]. Wan et al. recently evaluated the roles of cir-ITCH (itchy E3 ubiquitin protein ligase) in the progression of lung cancer cells using a Taqman-based qPCR method, and the results showed that the expression of cir-ITCH was decreased in two different cancer cell lines, A549 and NIC-H460. The results of mechanism studies showed that ectopic expression of cir-ITCH could facilitate ITCH expression via sponging miR-214 or miR-7, further leading to the inhibition lung cancer cell proliferation. Interestingly, in turn, both miR-214 and miR-7 could degrade cir-ITCH through binding to the ITCH sequences of cir-ITCH [52], supporting miRNA-mediated circRNA degradation (Figure 3). Results above indicated that, by interfering with cancers-associated-miRNAs, circRNAs are differentially expressed in lung cancer and involved in tumor formation.

## circRNAs AND HEPATOCELLULAR CARCINOMAS

The advent of RNA-seq or microarray initially suggested that circRNAs could be novel targets for clinical treatment in hepatocellular carcinomas (HCC). Furthermore, circRNA and its relative mRNA or miRNAs could co-exist in the same HCC patient and cooperate closely to promote carcinogenesis [53-55]. Currently, gene expression microarray has been regarded as a feasible and helpful method to recognize the molecular signatures, including circRNAs. Shang et al. used microarray analysis to investigate the circRNA expression in HCC and showed that the global expression profile of circRNAs between HCC tissue and normal liver tissue is significantly different. Among these alternative circRNAs, hsa_circ_0005075, located on chromosome 1, exhibits significantly downregulated expression and shows good potential for the diagnosis of HCC. Mechanistically, hsa_circ_0005075 acts as the sponge of miR-23b-5p to regulate the circRNA-miRNA-mRNA networks involved in liver cancer development [56]. Another low expressed circRNA, hsa_circ_0001649, derived from exons 26-29 of SHPRH (SNF2 histone linker PHD RING helicase) gene, is negatively associated with tumor size and occurrence. Knockdown of hsa_circ_0001649 could promote the expression of matrix metalloproteinases, further enhancing cancer cell metastasis [57]. Through a circRNA microarray analysis, one upregulated circRNAs, circRNA_100338, was recently identified as another potentially valuable biomarker for hepatitis B-related HCC. Functioning as an endogenous sponge for miR-141-3p, circRNA_100338 expression could enhance metastatic progression and shorten survival time [58]. Moreover, these differentially expressed circRNAs exhibit HCC-stage specific properties [59], further suggesting an independent role for the prognosis prediction of HCC. Through analyzing the expression profile of human circRNAs in HCC tissues, Han et al. identified circMTO1 as the significantly downregulated circRNA in HCC tissues. The results from this group suggested that circMTO1 could enhance the HCC cell proliferation and invasion by sponging miR-9 and increasing p21 level. Further Kaplan-Meier survival study revealed that the HCC patients with low circMTO1 expression have a shorter survival time [54]. Yu’s group executed qPCR analysis of circRNAs in 66 HCC tissues and reported a carcinogenic circRNA, ciRS-7, which promotes HCC cell proliferation by targeting miR-7 and elevating the expression of the direct target gene PIK3CD (phosphatidylinositol 3-kinase catalytic subunit delta) [60]. However, as measured by qPCR,, Xu et al. have showed no obvious difference in ciRS-7 expression in 108 pairs of HCC and matched non-tumor tissues [61]. These contradictory findings may reflect the inconsistent sample capacity. What–s more, as ciRS-7 contains more than 70 binding sites for miR-7 [62], these discrepant findings may also be due to the different down-stream factors of miR-7 influenced by ciRS-7 in different group of characters. Therefore, it is of importance to explore the detailed function and mechnism of ciRS-7 in pathophysiology and treatment of different HCC patients.

## circRNAs AND COLORECTAL CANCERS

As potential predictive or diagnostic biomarkers for colorectal cancers (CRC), circRNAs are attracting increasing research attention. Moreover, the activities of these molecules as gene expression regulators or miRNA sponges have been associated with CRC progression and chemoradiation senstitivity, with high sensitivity and specificity [63-65]. The differential expression profiles of circRNAs in 3 paired CRC cancerous and normal tissues indicated the significant upregulation (n=136) or downregulation (n=243) of 2608 circRNAs in CRC samples (fold changes > 2). Moreover, circ-BANP, generated from exons 5-11 of the BANP gene, is the most upregulated circRNA, and knockout of circ-BANP obviously reduced the proliferation rates of HT29 and HCT116 CRC cells by attenuating Akt/PKB (protein kinase B) phosphorylation [66]. Using unsupervised hierarchical clustering analysis (UHCA) technology, Guo et al. identified the most upregulated circRNA, has_circ_0000069, in 6 paired CRC cancerous and normal tissues and further verified its expression level in cancer cells using qPCR. Function analysis suggested that has_circ_0000069 could promote cell proliferation and metastasis in HT29 CRC cells [67]. Similarly, Hsiao’s group showed that elevated circCCDC66, derived from exons 6-11 of CCDC66, is associated with the poor prognosis of CRC. Mechanistically, after hypoxic stress, accumulated circCCDC66 could protect a specific group of oncogenes, such as the MYC gene, from interactions with miR-33b, miR-93, and miR-185, leading to the proliferation and metastasis of HT29 and HCT116 cells [68]. The overexpression of has_circ_001569 in CRC cells also functions as a miR-145 sponge and promotes the expression of its targets genes E2F5 (E2F transcription factor 5) and BAG4 (BCL2 associated athanogene 4) [69]. High expression of has_circ_0020397 could positively influence the cell viability and invasion via promoting the expression of miR-138 target genes, telomerase reverse transcriptase (TERT) and programmed death-ligand 1 (PD-L1) [70]. Apart from these markedly upregulated circRNAs, the global reduction of the abundance of circRNAs, including hsa_circ_001988, has been negatively associated with the proliferation index, and these molecules may also represent potential novel targets for CRC therapy [71, 72]. These studies clearly suggest the clinical relevance of circRNAs as promising biomakers, since their expression is able to distinguish CRC tissues from the adjacent non-tumor tissues. Moreover, these differentially-expressed circRNAs afore-mentioned may also be recognized as molecular biomakers in assessing the curative effect of chemoradiotherapy on CRC patients. In addition, deep RNA-seq analysis of the exosomes from CRC cells showed that hundreds of candidate circRNAs could be transferred into exosomes [73], suggesting that extracellular-vesicle derived circRNAs in CRC patients may also represent potential targets for therapeutic interventions in the future.

## circRNAs AND GASTRIC CANCERS

CircRNA profiles and circRNA-miRNA-mRNA interactions in gastric cancers (GC) have recently been discussed, and studies have demonstrated that differentially expressed circRNAs could serve as novel and stable makers for the diagnosis and progression of GC patients [74, 75]. In a recent study, based on microarray chip technology, Sui et al. screened 467 significant differences (>2-fold changes, p-values<0.05) in circRNA expression, with 214 upregulated and 253 downregulated molecules identified in 8 GC tissues and adjacent tissue samples. In-house miRNA target prediction software revealed miRNA-binding sites in 69 differentially expressed circRNAs [76]. Using a circRNA microarray, Huang et al. confirmed only one significantly differently down-expressed circRNA, circRNA-0026, in GC tissues. Biomathematical analyses indicated that circRNA-0026 regulates RNA transcription, RNA metabolism, and other biological functions by adsorbing cancer-associated miRNAs [77]. Thereby, these differentially expressed circRNAs could sponge corresponding miRNAs to modulate the expression of their target genes. For example, the upregulated circPVT1, derived from the PVT1 locus on chromosome 8q24, promotes GC progression via acting as a sponge for the miR-125 family [78]. Additionally, the low expressed hsa_circ_0000096 could regulate the expression levels of cell cycle- or metastasis-correlated proteins, affecting the growth and migration of GC cells [79]. Another downregulated circRNA_100269 could target miR-630, resulting in GC cell growth suppression [80]. These data laid the foundation to investigate the attractive clinical significance of distinct circRNA candidates for GC pathobiology. According to the data obtained from CircBase database, Shao et al. showed that has_circ_0001895 is downregulated in GC cells and significantly associated with histological type and CEA expression [81]. Tian et al. demonstrated the negatively association between has_circ_0003159 expression and clinicopathological factors of GC patients [82]. Similarly, another two typically downregulated circRNAs, hsa_circ_002059 and hsa_circ_00001649, have been associated with clinicopathological characteristics, such as differentiation, TNM stage, gender, etc. [83, 84]. In addition, Zhang et al. generated a circRNA-based classifier to assess the recurrence risk after radical surgery using four significantly differentially expressed circRNAs in GC samples. These studies showed that this predictive tool could effectively distinguish the high-risk patients with recurrence [85].

## circRNAs AND HEMATOLOGICAL MALIGNANCIES

**Figure 4:**
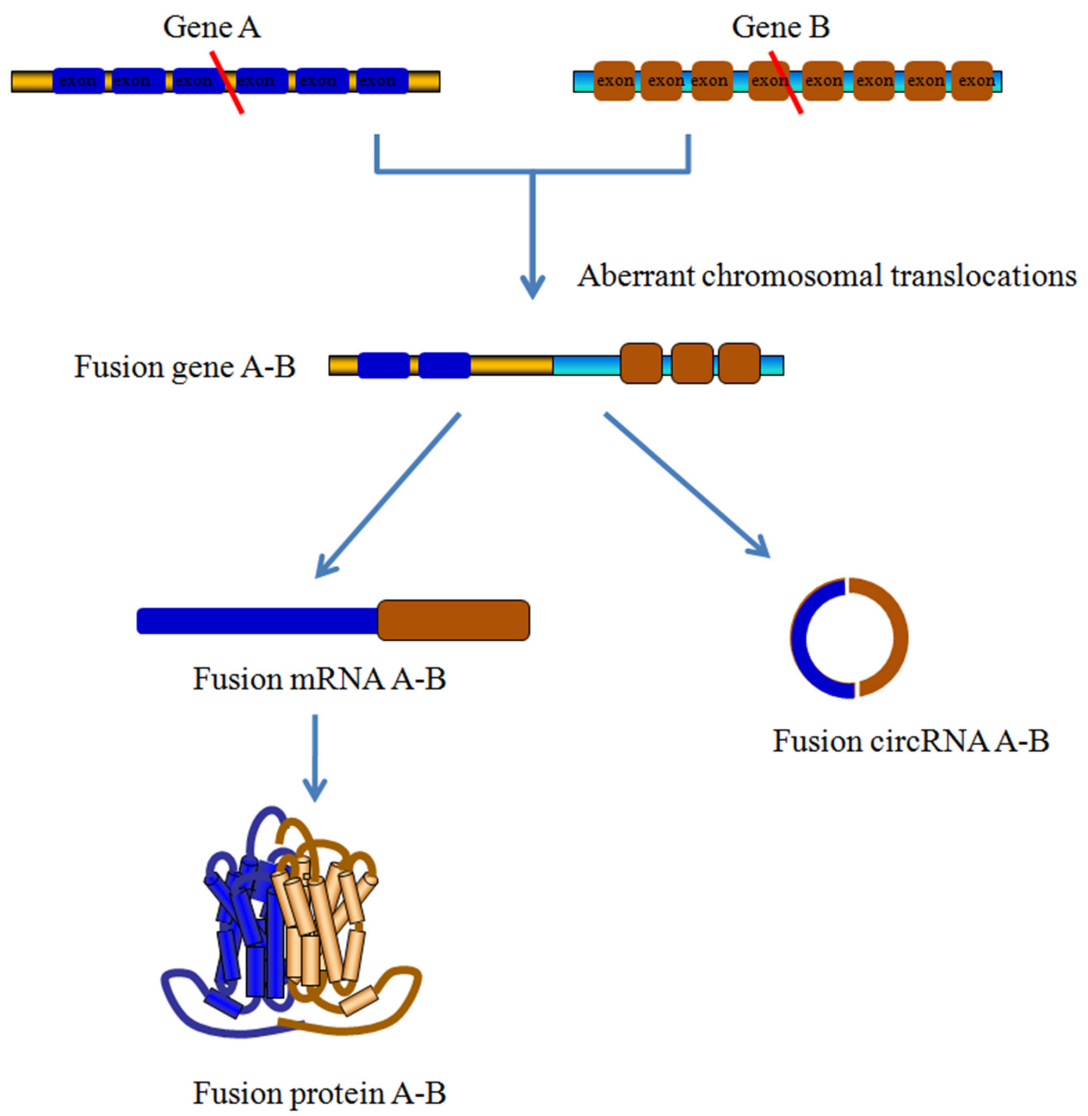
Fusion-circRNAs derived from aberrant chromosomal translocations Cancer-related chromosomal translocations could produce linear fusion mRNA and fusion circRNAs (f-circRNAs). The formation of f-circRNAs is shown here. Briefly, after the chromosomal translocation of Gene A and Gene B, the fusion and backsplice junctions of one exon from Gene A and another exon from Gene B result in the formation of f-circRNA. Meanwhile, corresponding linear fusion mRNA can be translated into functional fusion proteins. Both fusion proteins and f-circRNAs involve in the cancer development and progression, especially the hematological malignancies.

Increasing evidence has demonstrated that fusion genes, encoded by aberrant chromosomal translocations, are undoubtedly involved in malignant hematological disorders. In addition, chromosomal rearrangements affect ncRNAs levels in tumor cells through complicated mechanisms, such as the direct generation of fusion circRNAs (f-circRNAs) [86] (Figure 4). Further studies have suggested that these cancer-associated fusion genes could give rise to multiple f-circRNAs, such as f-circPR and f-circM9_1, which show pro-proliferative and proto-oncogenic properties [87]. Using ACFS software to analysis the data from single- and paired-ended RNA-Seq, You et al. identified a several differentially expressed circRNAs between AML and acute promyelocytic leukemia (APL) patients. The ACFS database could further verify the f-circRNAs in leukemia tissues with high accuracy and a low false discovery rate [88]. These data support the notion that f-circRNA plays an active function in favoring the progression of hematological malignancies. Apart from the f-circRNAs, Li et al. identified another remarkably downregulated circRNA, has_circ_0004277, in a cohort of 115 human acute myelocytic leukemia (AML) samples. The findings from this group indicated that standard chemotherapy could recover the expression of has_circ_0004277 and its corresponding linear isoform WDR37 (WD repeat domain 37), suggesting that elevated has_circ_0004277 may affect the chemotherapy response [89].

## circRNAs AND BLADDER CARCINOMAS

Competing endogenous RNAs (ceRNA) have recently been identified specifically upon cancer onset and development, reflecting the pivotal effect of these molecules on the expression of cancer-associated miRNAs (Figure 2). A recent study analyzing the ceRNA network from the microarray data of bladder cancer (BC) and para-cancer tissues showed that uniform MREs were observed in both circRNAs and lncRNAs. For example, lncRNA H19 and circRNA MYLK competitively bind miR-29a-3p via its corresponding MREs, resulting in an increase of miR-29a-3p target genes. Moreover, through regulating the expression of miR-29a, circRNA MYLK plays a key role in accelerating cancer progression of BC patients [90, 91]. In addition, Zhong et al. identified 469 dysregulated cercRNAs (285 upregulated and 184 downregulated) in BC compared with normal samples. Bioinformatics approaches preliminarily showed that these abnormally expressed circRNAs, particularly circTCF25, participate in multiple cancer-associated signaling pathways, functioning as valuable biomarkers for the diagnosis and therapy of BC [92]. However, the detailed mechanisms and functions of these differentially expressed circRNAs in BC development require further discussion.

## circRNAs AND ESOPHAGEAL CELL CARCINOMAS

Function analysis of circRNA in esophageal cell carcinomas (ESCC) has provided much evidence supporting the exploitation of RNA-based treatment strategy. Expression profile analysis and bioinformatics methods revealed that clusters of circRNAs are aberrantly expressed in radioresistant KYSE-150R esophageal cancer cells compared with parental KYSE-150 cells (fold-change ≥2.0 and P < 0.05). Gene ontology (GO) analysis further demonstrated that the genes in the Wnt signaling pathway are the main targets of these candidate circRNAs [93]. In addition, a highly expressed circRNA, has_circ_0067934, has been associated with poor differentiation and TNM stage in ESCC patients. Silencing of hsa_circ_0067934 using siRNA could alleviate the proliferation and migration of ECA-109 and TE-13 ESCC cells [94]. Taken together, these findings indicated key roles for circRNAs in ESCC development and treatment.

## circRNAs AND BREAST CANCER

Latest studies have shown that circRNAs could be the important regulatory molecules for the development of breast cancer [95], one of the most frequently occurring cancers worldwide [96]. Using the Arraystar Human circRNA Array to construct a genome-wide circRNA profile, researchers have found about 1155 differently expressed circRNAs (715 up-regulated and 440 down-regulated) in breast cancer tissues. Then they choose three elevated circRNAs (has_circ_103110, has_circ_104689 and has_circ_104821) and three down-regulated circRNA (has_circ_006054, has_circ_100219 and has_circ_406697) as the promising candidates for the diagnosis of breast cancer. The areas under the curve (AUC) of has_circ_100219 was 0.78, indicating the highest diagnostic accuracy [97]. Liang et al. identified the hypoxic-inducible factor 1 alpha (HIF1α) associated cricRNA, circDENND4C, and found that knockdown of circDENND4C by siRNAs resulted in decreased proliferation of breast cancer cells MCF7 and MDA-MB-231 in hypoxic environment. The clinical relevance analysis indicated a positive correlation between circDENND4C level and the tumor size [98]. Through investigating the differently expressed circRNAs in human breast cancers, another group has found that circ-ABCB10, the most significantly up-regulated circRNA, could facilitate the cancer proliferation and progression by sponging miR-1271 [99]. Meanwhile, Yang et al. demonstrated the tumorigenic capacity of circ-Amotl1, an important circRNA highly expressed in breast cancer tissues and cells. Mechanistically, ectopic expression of circ-Amotl1 mainly triggered the nuclear translocation of proto-oncogene c-Myc, enhancing the c-Myc stability and furtherly increaseing the c-myc targets [100]. Taken together, all these studies reveal the important regulatory role of circRNAs for breast cancer pathogenesis.

## circRNAs AND PANCREATIC DUCTAL ADENOCARCINOMAS

Accumulating studies have reported a cluster of circRNAs in the malignant behavior of pancreatic ductal adenocarcinomas (PDAC). The abundance of aberrantly expressed circRNAs, identified using microarray technology, have been implicated in carcinogenesis, providing novel insight into PDAC biology [101, 102].

## DATABASES FOR circRNA DETECTING IN CANCER CELLS

In recent years, several algorithms have been developed to annotate and identify circRNAs from RNA-seq data obtained from cancer samples (Table 2). The CircNet database provides comprehensive information on circRNAs, including genome sequences and loci, and more importantly, this database also provides information for integrated ciRNA-miRNA-mRNA networks [29]. The UROBORUS database, developed by Song et al., contains the low expressed circRNAs from the total RNA-seq without RNase R treatment [46]. CIRCexplorer tools can detect candidate circRNAs in breast cancer tissues with high accuracy and good sensitivity, but it requires their gene annotation [41]. ACFS software proposes a fast and quantitative detection method to identify circRNAs from single- and paired-ended RNA-seq datasets of leukemia. In addition, compared with other databases, ACFS shows the highest accuracy and lowest false discovery rate [88]. Circ-Seq is a comprehensive algorithm used to define coincident circRNAs from public breast cancer samples provided by The Cancer Genome Atlas (TCGA) and Genotype-Tissue Expression Project (GTEx) [103]. The website circBase has offered a method to freely access and download the merged and unified data sets of cancer related circRNAs [83, 104]. The study by Ghosal et al. introduced another detection tool, Circ2Traits, for circRNAs potentially associated with human diseases, like cancers. Using this algorithm, researchers could also get the cancer associated single nucleotide polymorphisms (SNPs) on circRNA loci [105]. StarBase v2.0, designed by Li’s group, systematically provides the circRNA–miRNA-mRNA and protein–circRNA interaction networks from large-scale data sets of normal tissues and cancer cells [106]. Other databases, such as PcircRNA_finder [107], CircInteractome [108], deepBase v2.0 [109], CIRI2 [110], et al. have not currently been used to screen circRNAs in cancerous samples, but these algorithms would offer more choices to compare and obtain more reliable results. Although these algorithms have their own advantages, problems, such as little overlap in prediction and no clear gold standard, should be addressed in the future [111]. Therefore, an important step for using these bioinformatics approaches is to adjust the search strategy appropriately to elevate the confidence with an appropriate threshold.

**Table 2:** Databases used for circRNAs detection in cancers tissues and cells

Databases	De novo?	Characteristics	URL	Refs
CircNet	No	Tissue-specific circRNA expression profiles; Integrated CiRNA-miRNA -mRNA networks.	http://circnet.mbc.nctu.edu.tw/	[29]
CIRCexplorer	No	Depending on the mapping strategy of aligners; CircRNAs annotation only.	http://yanglab.github.io/CIRCexplorer/	[41]
UROBORUS	No	Total RNA-seq data with no poly(A) depletion or RNase R treatment; Detecting lower expressed circRNAs; Lower false positive rate.	http://uroborus.openbioinformatics.org/en/latest/	[46]
Acfs	Yes	Quantification and fast; Highest F1 accuracy; Lowest false discovery rate; Identify fusion circRNAs.	https://github.com/arthuryxt/acfs	[87]
Circ-Seq	No	Genomic annotation; Fully automated; Running in a multi-threaded cluster.	http://bioinformaticstools.mayo.edu/research/circ-seq/	[102]
circBase	No	Downloading freely the merged and unified data sets of circRNAs	http://www.circbase.org/	[103]
Circ2Traits	No	A disease-circRNA association database Identifying diseases associated SNPs on circRNAs loci	http://gyanxet-beta.com/circdb/	[104]
starBase v2.0	No	Providing comprehensive circRNA–miRNA-mRNA and protein–circRNA interaction networks	http://starbase.sysu.edu.cn/	[105]

## DISCUSSION AND CONCLUSION

The recent revelation of the widespread existence of circRNAs sheds an attractive light on the research and therapy of human diseases. Mechanistic and functional studies have revealed that circRNAs are no longer considered useless junk sequences in genomes. Although current knowledge of the biological effect of circRNAs remains limited, the exploration of the roles of circRNA in cancer biology could bring oncologist closer to the tumor cure. Particularly, in recent years, advancements in molecular medicine have helped to predict the potential for circRNAs serving as profound markers for cancer diagnosis and prognosis [112]. Currently, the abundance of natural compounds has emphasized the anti-tumor effects of these molecules via different signaling pathways [113]. Exploring the therapeutic agents that interfere with circRNAs or their effectors could reveal beneficial strategies to eradicate cancer cells.

The clinical potential for the use of circRNAs as diagnostic biomarkers and predictive targets for cancers continues to expand. Recent systemic deep RNA-seq studies have verified that the differential expression of circRNAs is well correlated with some clinicopathological features, such as tumor TNM stages, recurrence, metastasis, etc [61, 85]. For example, the upregulated level of circPVT1 in GC tissues could be served as an independent prognostic marker for overall survival and disease-free survival of cancer patients [78]. Another candidate circRNA, hsa_circ_0004018, has been proved to harbor the tumor stage-specific expression features in diverse chronic liver diseases [59]. Meanwhile, with the AUC of 0.75 in tissues and 0.60 in plasma, respectively, hsa_circ_0000190 showed better sensitivity and specificity than commonly used biomarkers such as CEA and CA19-9 for GC patients [30]. In addition, the specimens for the circRNAs detection should be easily acquired from the cancer patients, including the blood, urine, saliva, etc. CircRNAs have been proved to be detected in the cell-free components of the body fluids, with benign extracellular stability [114]. Reports on circRNAs in exosomes might represent a novel aspect for life sciences [42]. Extracellular vesicles, including exosomes, isolated from the blood and other biofluids of cancerous patients, contain a specific cargo of RNA and protein species, which regulate various cell bio-behaviors in human diseases with both diagnostic and therapeutic implications [115]. Recent studies have demonstrated that cancer-associated circRNAs could be identified in tumor-derived exosomes in patients with colon cancer or leukemia [73, 87]. Thus, the circRNAs in body fluids could be used as promising pathognomonic markers for different types of cancers, particularly the cancers for which biopsies are difficult to obtain. Though these above-mentioned studies have showed preliminary findings about the clinical significances of circRNAs, much clinical relevance still remains to be clarified about the roles of circRNAs before coming full article.

In addition, other cancer-related biological pathways, such as immunocyte aging within the immune system, could combat or sustain cancer development and progression. Recently, Wang et al. analyzed the comprehensive circRNA profile in individuals undergoing immunosenescence, and showed that circular RNA100783 plays an important role in immune T cell aging [116]. Further examination revealed that the expression of circRNAs could be regulated by several Toll-like receptors [117], which induce the appropriate immune responses in cancer patients [118]. Although the detailed mechanisms of circRNAs in immune regulation remain largely unclear, understanding the potential functions of circRNAs in immune responses is of significant relevance to cancer therapy. Thus, extensive studies should focus on the role of circRNAs in certain aspects of cancer biology, such as immunity regulation signaling, which may represent another useful therapeutic strategy for human cancers.

## PERSPECTIVE

As a kind of newly discovered endogenous ncRNAs, circRNAs have been proved to involve in multiple cellular processes of tumorigenesis by regulating gene expression at the transcriptional or post-transcriptional level. Thousands of well-expressed circRNAs are abundant and stable in cancer cells, suggesting that they might be the key factors for cancer occurrence and development. Especially, numerous reports have shown that the circRNAs-miRNA-mRNA axis plays a pivotal role in several cancer-associated biological behaviors, such as proliferation, angiogenesis and metastasis [51, 119, 120]. Meanwhile, by interacting with miRNAs, circRNAs also have the potential of impacting a variety of signaling pathways in cancers, such as vascular endothelial growth factor A (VEGFA) signaling pathway [91], large tumor suppressor kinase 1 (LATS1) [121], Src family tyrosine kinases (SFKs) [122] and so on. More importantly, the specificity and sensitivity of circRNAs are helpful to make them be possible biomakers, like the hsa_circ_0004018 in hepatocellular carcinoma [59]. With the progress of molecular biological technique and bioinformatics, other novel circRNAs that were identified recently might also serve as promising biomakers.

However, in the clinical setting, the detailed functions of circRNAs in human cancers need to be further explored. And several outstanding issues of circRNA study to focus on in the future are as follows: (1) Although identified in the 1980s [123], the information of circRNAs in human diseases, especially cancers, is still limited. There is a need to screen and recognize new candidate circRNAs that can serve as promising therapeutic targets, not just the diagnostic and prognosis biomarkers. (2) To data, rapid development of technology have made RNA-seq to be the wide method to identify circRNAs in cancer samples and cells accurately. However, as the next generation sequencing is expensive and time-consuming, the use of circRNAs for the detection of cancers needs to be detected in much easier and cheaper methods. For example, two other methods, qPCR and *in-situ* hybridization, have been proved to be more sensitive and specific for detecting circRNAs [124]. (3) The potential of circRNAs as valuable makers for cancer diagnose and prognosis is needs to be further well-explored in the future. Because most studies above-mentioned lack the tangible proofs and well-established standard supporting the reasonable clinical application.
